# Diffuse filiform polyposis of the small intestine without inflammatory bowel disease

**DOI:** 10.1186/1477-7819-12-396

**Published:** 2014-12-23

**Authors:** Jin-Wei Jiang, Guan-Yu Wang, Yi-Ping Zhu, Ren-Biao Chen, Ze-Qin Zhang, Yu-Jie Zhang

**Affiliations:** Department of General Surgery, Sir Run Run Shaw Hospital, School of Medicine, Zhejiang University, East Qingchun Road, Hangzhou, 310016 Zhejiang Province China; Department of Radiology, Sir Run Run Shaw Hospital, School of Medicine, Zhejiang University, Hangzhou, 310016 Zhejiang Province China

**Keywords:** Abdominal angiographic embolization, anemia, Digital subtraction angiography, Filiform polyposis, melena, Worm-like polyps

## Abstract

Filiform polyposis is a rare disease, which typically occurs in patients with inflammatory bowel disease. We report a case of filiform polyposis occurring in a 56-year-old man with no history or evidence of inflammatory bowel disease. The patient’s main symptoms were melena and anemia. We performed an emergency exploratory laparotomy, in which we observed worm-like polyps spread almost along the entire small intestine, and a partial resection of the small intestine to treat bleeding in the bowel was carried out. Two days later, the patient was noted to have melena again, and we performed an abdominal angiographic embolization, successfully stopping the bleeding. Histologic evaluation of the excised specimen revealed chronic inflammatory cells within the lamina propria without hyperplastic or adenomatous epithelial changes. Although the surgery was very successful, careful management of the patient was required, owing to the great risk of re-bleeding.

## Background

Filiform polyposis is an uncommon entity manifested by multiple slender worm-like projections, histologically characterized as submucosal fibrovascular accentuation within normal mucosa [1]. Filiform polyposis is mostly asymptomatic and incidentally diagnosed on colonoscopy. However, patients may present with a variety of symptoms, including anemia, weight loss, cramping abdominal pain, and diarrhea. The condition can also produce obstruction and intussusceptions, in addition to bleeding [2, 3]. The vast majority of cases occur in the setting of inflammatory bowel disease, particularly in patients with ulcerative colitis. Crohn’s disease can present as a giant mass lesion containing inflammatory polyposis [2]. The pathogenesis of filiform polyposis is uncertain. However, long-term inflammation of the colonic mucosa during chronic inflammatory bowel disease with alternating periods of ulceration and healing may lead to the formation of finger-like projections [4]. Although filiform polyposis typically occurs in patients with inflammatory bowel disease, cases have been documented in patients without inflammatory bowel disease. Here, we report a case of filiform polyposis of the small intestine in a 56-year-old man with no history or evidence of inflammatory bowel disease.

## Case report

**Figure 1:**
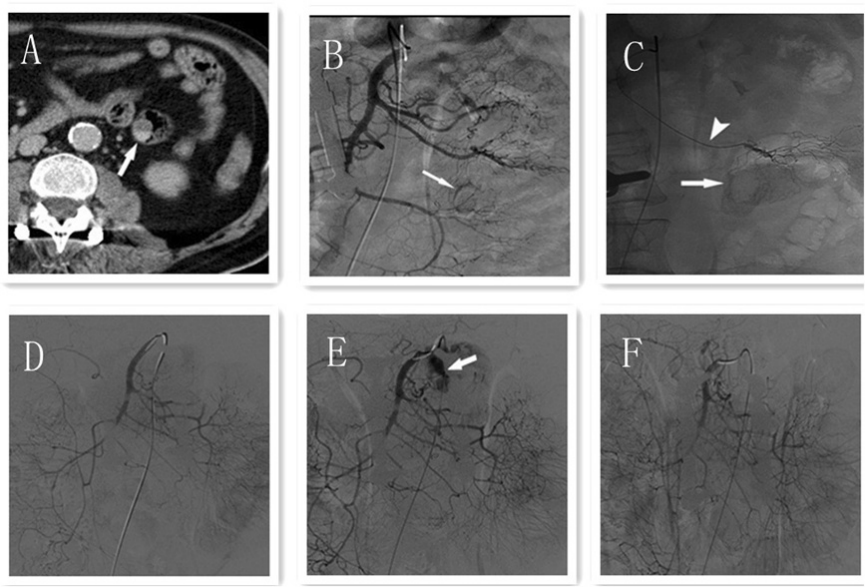
**Abdominal contrast-enhanced computed tomography and digital subtraction angiography of superior mesenteric artery of the patient. (A)** Abdominal contrast-enhanced computed tomography demonstrated a round abnormal enhancement (arrow) in the small intestinal lumen within the left upper quadrant. **(B)** Digital subtraction angiography of superior mesenteric artery demonstrated a rim-like staining (arrow) in the left upper quadrant. **(C)** A microcatheter was inserted into the feeding artery (arrow head); rim-like staining (arrow) was revealed after contrast medium injected in the microcatheter. **(D)** Digital subtraction angiography of the superior mesenteric artery demonstrated no contrast medium extravasation when the blood pressure was 90/70 mmHg. **(E)** Digital subtraction angiography of the superior mesenteric artery demonstrated contrast medium extravasated into the intestinal tract (arrow) when the blood pressure was raised to 120/80 mmHg. **(F)** Digital subtraction angiography of the superior mesenteric artery demonstrated no contrast medium extravasation after injection of 5 ml suspension of gelatin sponge particles (500 μm) and contrast medium.

**Figure 2:**
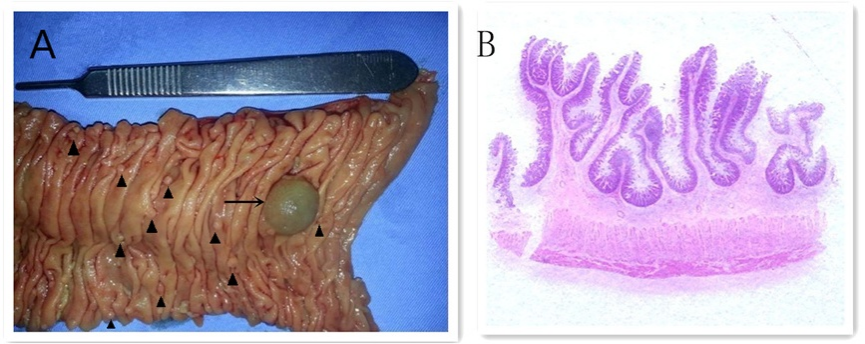
**Resected small intestine and histological section of the filiform polyps. (A)** Gross appearance: numerous worm-like filiform polyps (arrow head) and 2-cm-diameter hematoma (arrow) are apparent. The filiform polyps spread throughout the small intestine, ranging in size from 0.2 cm to 0.5 cm. Bleeding points could be found after the hematoma was removed. **(B)** Histological sections of the filiform polyps.

**Figure 3:**
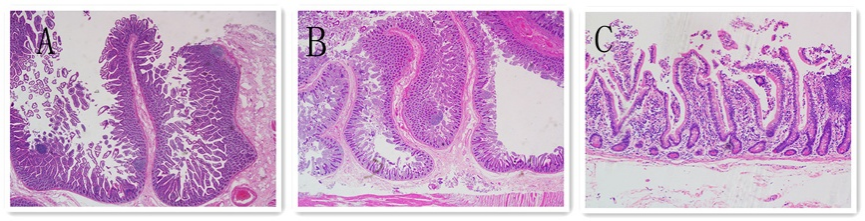
**Microscopic appearance of the filiform polyps of the resected small intestine. (A,B)** H & E staining, ×20; **(C)** H & E staining, ×200. The polyps appear as slender finger-like stretching projections and are covered by histologically normal colonic mucosa. The stalks of the polyps consist of submucosal fibrovascular components. Mild chronic inflammatory cells could be seen in the lamina propria without hyperplastic or adenomatous epithelial changes.

A 56-year-old man initially presented at our hospital complaining of having had melena for half a month. He did not have any personal or family history of bowel polyps, bowel cancer, or inflammatory bowel disease. He denied having chills, fever, nausea, vomiting, diarrhea, or abdominal pain. The flaring-up of melena was sudden and the patient was admitted, owing to tarry stools and melena. Results of a physical examination were unremarkable apart from an anemic appearance. A complete blood count test revealed anemia (hemoglobin level 4.8 g/dl) and the prothrombin time was 15.6 s (normal range, 11.5 to 14.5 s). Liver function, kidney function, and inflammatory indexes were noted to be normal. Abdominal contrast-enhanced computed tomography demonstrated a round abnormal enhancement in the lumen of the small intestine lumen in the left upper quadrant (Figure 1A), at about the level of L3, raising suspicion of tumors or vascular lesions. Computed tomography also demonstrated a hematocele in the small intestine and colon (especially in the ileocecum). Digital subtraction angiography revealed contrast agent staining in the left upper quadrant (Figure 1B) and a microcatheter was placed in the feeding artery (Figure 1C). Gastroscopy indicated multiple duodenal polyps. Colonoscopy was unsuccessful, owing to the presence of a hematocele. Subsequently, the patient underwent a partial resection of the small intestine. Worm-shaped polyps and small hematomas were noted; these were spread along almost the entire small intestine, and they could not be completely resected. To identify the bleeding section of the bowel, we injected methylene blue into the indwelling catheter, staining an approximately 30 cm section of jejunum. The stained section was subsequently resected. Grossly, the resected specimen revealed multiple worm-like polyps, which ranged in size from 0.2 cm to 0.5 cm; two 2-cm-diameter hematomas were also noted (Figure 2). The round, abnormal enhancement demonstrated on computed tomography was considered to be a hematoma. Histologically, the polyps consisted of normal mucosa characterized by nonspecific inflammatory changes (Figure 3). Two days after the surgery, the patient had melena again. We subsequently performed an abdominal angiographic embolization. Throughout the course of this procedure, no bleeding points were noted. There was no contrast medium extravasation when the blood pressure was 90/70 mmHg (Figure 1D). We raised the blood pressure, and digital subtraction angiography demonstrated contrast medium extravasated to the intestinal tract (Figure 1E). A 5 ml suspension of gelatin sponge particles was injected and successfully stopped the bleeding (Figure 1F). The patient was informed that he had a high risk of re-bleeding owing to polyps and careful management was required. After the operation, the patient recovered smoothly, the patient’s hemoglobin level increased to 10.7 g/dl and the patient was discharged from the hospital. No recurrent melena or bleeding was reported at the last follow-up in January 2014.

## Discussion and conclusions

The term filiform polyposis was first defined by Appelman, who used it to describe a syndrome involving the radiographic appearance of numerous long slender worm-like or filiform defects in the colon, along with a normal haustral pattern [1]. Other terms, such as post-inflammatory polyps, giant pseudopolyposis, and giant inflammatory polyposis, have been used in the literature to describe this kind of polyposis [5–10]. Although esophageal, gastric, and intestinal filiform polyps have been described, the colon remains the most involved site, and the sigmoid colon is the commonest location [11]. A complete colonoscopy is important, because polyposis can extend as far as the cecum [2].

On microscopic examination, the polyps are lined by normal, edematous, or superficially ulcerated colonic mucosa [11]. Filiform polyposis usually has a thin, straight shape resembling the stalks of polyps without the heads [2]. The polyps usually range in size from 1.5 to 3.0 cm in length. Some are long, slender, worm-like or finger-like projections that can extend up to 9 cm in length [12].

In patients with inflammatory bowel disease, filiform polyposis was previously generally thought to be associated with the post-inflammatory reparative process [13, 14]. However, about 21 similar cases of filiform polyposis in patients without a history of inflammatory bowel disease (7 women, 14 men) have been reported at the time of writing [11]. Filiform polyposis may be sequelae of prior bacillary dysentery, necrotizing enterocolitis, enema-induced colitis, uretero-sigmoidostomy, stercoral ulcer, Langerhans cell histiocytosis X, or colonic tuberculosis [2]. Therefore, some authors suggest that the pathogenesis of filiform polyps may not be related to a post-inflammatory reparative process but instead to a hamartomatous process because they have recorded observations of neuromuscular and fibrovascular hyperplasia or disarray [15].

The polyps may be localized, or they may diffusely involve the colon. Diffuse colonic filiform polyposis may endoscopically mimic familial adenomatous polyposis. Furthermore, numerous conglomerated polyps might give the appearance of a fungating mass; such a mass has been confused with cancer in colonoscopy and radiology studies. In some cases, the polyps are difficult to distinguish from villous adenomas, necessitating a biopsy or polypectomy to establish an exact diagnosis [2]. Generally, there is no definite evidence that filiform polyposis itself represents a precancerous condition, while adenomatous polyps possess malignant potential [4].

The treatment of biopsy-confirmed inflammatory polyps may remain conservative as long as these lesions are asymptomatic. If the polyp is pedunculated, easily accessible, and can be removed with minimal risk of hemorrhage, then it is preferable to remove it [2]. Filiform polyposis alone is not an indication for surgical resection; however, complications, such as acute massive hemorrhage or intestinal obstruction, may necessitate surgical intervention [16]. When patients with inflammatory polyposis require surgical management, it is important to evaluate the margins of resection, because inflammatory polyposis can recur in the presence of acute inflammation or residual disease at the resected margins [17]. Nevertheless, in our case, we injected methylene blue into the indwelling catheter to identify bleeding sections of the bowel, as the polyps were spread along almost the entire small intestine, and it is not advisable to resect the entire small intestine. Recurrence was therefore very likely and regular follow-up examinations were recommended to the patient.

In addition, our second digital subtraction angiography did not initially reveal any bleeding points. We increased the patient’s blood pressure by injecting adrenaline; only then did the bleeding points become apparent. We hypothesized that low blood pressure would lead to a decrease in bleeding volume. Raising the blood pressure proves to be a good choice when intestinal bleeding points cannot be found in patients suspected of bleeding. Surgeons, however, should be careful in the course of treatment, as massive hemorrhage remains a great risk.

## Consent

Written informed consent was obtained from the patient for publication of this case report and accompanying images. A copy of the written consent is available for review by the editor-in-chief of this journal.

## Abbreviations

H & E: hematoxylin and eosin.
